# Biopsy-Confirmed Serofrequency of Celiac Disease among Patients with Disorders of Gut-Brain Interaction in Japan: A Multicenter Cross-Sectional Cohort Study

**DOI:** 10.1159/iid/adeag003

**Published:** 2026-09-01

**Authors:** Masaaki Higashiyama, Chie Kurihara, Akiko Shiotani, Takeshi Mizukami, Mikihiro Fujiya, Akihito Nagahara, Seiji Futagami, Hiroyuki Imaeda, Eikichi Ihara, Katsunori Iijima, Shuji Terai, Toshio Uraoka, Yasuhiro Fujiwara, Akira Andoh, Naoki Ohmiya, Hiromi Kataoka, Tadayuki Oshima, Takayuki Matsumoto, Hiroshi Nakase, Hirotake Sakuraba, Mamoru Watanabe, Kazushige Uchida, Hidekazu Suzuki, Masanao Nakamura, Yoshikazu Kinoshita, Govind K. Makharia, Ryota Hokari

**Affiliations:** a Department of Internal Medicine, National Defense Medical College, Saitama, Japan; b Department of Gastroenterology and Hepatology, Kawasaki Medical School, Okayama, Japan; c Department of Endoscopy Center, NHO Kurihama Medical and Addiction Center, Yokosuka, Japan; d Division of Gastroenterology, Department of Internal Medicine, Asahikawa Medical University, Hokkaido, Japan; e Departments of Gastroenterology, Juntendo University Faculty of Medicine, Tokyo, Japan; f Division of Gastroenterology, Nippon Medical School, Tokyo, Japan; g Department of Gastroenterology, Saitama Medical University, Saitama, Japan; h Department of Medicine and Bioregulatory Science, Graduate School of Medical Sciences, Kyushu University, Fukuoka, Japan; i Department of Gastroenterology, Akita University Graduate School of Medicine, Akita, Japan; j Division of Gastroenterology and Hepatology, Graduate School of Medical and Dental Sciences, Niigata University, Niigata, Japan; k Department of Gastroenterology and Hepatology, Gunma University Graduate School of Medicine, Maebashi, Japan; l Department of Gastroenterology, Osaka Metropolitan University Graduate School of Medicine, Osaka, Japan; m Department of Medicine, Shiga University of Medical Science, Otsu, Japan; n Department of Advanced Endoscopy, Fujita Health University School of Medicine, Aichi, Japan; o Department of Gastroenterology and Metabolism, Nagoya City University Graduate School of Medical Sciences, Nagoya, Japan; p Department of Gastroenterology and Hepatology, Hyogo Medical University, Hyogo, Japan; q Division of Gastroenterology and Hepatology, Department of Internal Medicine, School of Medicine, Iwate Medical University, Iwate, Japan; r Division of Gastroenterology and Hepatology, Department of Internal Medicine, Sapporo Medical University School of Medicine, Sapporo, Japan; s Department of Gastroenterology, Hematology and Clinical Immunology, Hirosaki University Graduate School of Medicine, Aomori, Japan; t Organoid Center, Graduate School of Medicine, Juntendo University, Tokyo, Japan; u Department of Gastroenterology and Hepatology, Kochi Medical School, Kochi University, Kochi, Japan; v Division of Gastroenterology and Hepatology, Department of Internal Medicine, Tokai University School of Medicine, Kanagawa, Japan; w Department of Gastroenterology and Hepatology, Nagoya University Graduate School of Medicine, Aichi, Japan; x Hyogo-prefectural Harima-Himeji General Medical Center, Himeji, Japan; y Department of Gastroenterology, All India Institute of Medical Sciences, New Delhi, India

**Keywords:** Celiac disease, Irritable bowel syndrome, Functional dyspepsia, IgA tissue transglutaminase

## Abstract

**Introduction:**

While celiac disease (CeD) is an emerging disease worldwide, its prevalence has traditionally been very low in Japan. However, there are no regional data available regarding CeD across Japan. In this study, we screened patients with disorders of gut-brain interaction (DGBI), including irritable bowel syndrome (IBS) and functional dyspepsia (FD), who were supposedly at a high risk for CeD. Subjects were enrolled from the Hokkaido area (northern Japan) to the Kyushu area (southern Japan) to determine the biopsy-confirmed serofrequency of CeD across Japan and its regional distribution.

**Methods:**

In this study, adult patients fulfilling the criteria for IBS or FD were recruited from the outpatient departments of 23 participating centers in Japan. Anti-tissue transglutaminase IgA (TTG) titers were determined for serological screening. Further histological studies were performed in patients with TTG levels >10 u/mL.

**Results:**

In total, 583 patients were enrolled in this study. There was only one subject with TTG >10 u/mL among the subjects with diarrhea-predominant IBS, who was histologically diagnosed with CeD of Marsh 3a. Biopsy-confirmed serofrequency was estimated to be 0.17% among DGBI in Japan. The regional distribution was not evaluated because of its rarity.

**Conclusion:**

It is noteworthy that no clustering of CeD cases in Japanese patients with DGBI was observed, and the overall frequency remained extremely low across all participating centers nationwide.

## Plain Language Summary

While celiac disease (CeD) is an emerging disease worldwide, its prevalence has traditionally been very low in Japan. However, there are no regional data available regarding CeD across Japan. In this study, we screened patients with disorders of gut-brain interaction (DGBI), including irritable bowel syndrome (IBS) and functional dyspepsia (FD), who were supposedly at a high risk for CeD. Adult subjects fulfilling the criteria for IBS or FD were enrolled from the Hokkaido area (northern Japan) to the Kyushu area (southern Japan) to determine the biopsy-confirmed seroprevalence of CeD across Japan and its regional distribution. Anti-tissue transglutaminase IgA (TTG) titers were determined for serological screening. Further histological studies were performed in patients with TTG levels >10 u/mL. In total, 583 patients were enrolled. There was only one subject with TTG >10 u/mL among the subjects with diarrhea-predominant IBS, who was histologically diagnosed with CeD of Marsh 3a. The biopsy-confirmed seroprevalence was estimated to be 0.17% among DGBI in Japan. The regional distribution was not evaluated because of its rarity. We concluded that the biopsy-confirmed seroprevalence of CeD in Japanese patients with DGBI in our multicenter study is 0.17%, implying that currently the prevalence is very low across Japan.

## Introduction

Celiac disease (CeD), an autoimmune enteropathy triggered by dietary gluten in genetically susceptible individuals, is emerging worldwide, with a prevalence rate estimated to be as high as 1% [1]. This phenomenal increase in recognition has been made possible by the advent of disease-specific and reliable serological tests [2], including anti-tissue transglutaminase IgA (TTG) antibodies.

In East Asian countries, including China, Mongolia, Korea, and Japan, CeD is still considered rare, likely due to the limited consumption of a gluten-containing diet as well as the lower prevalence of the CD-susceptible HLA types HLA-DQ2 and -DQ8 [3, 4]. Among the general population in Shimane Prefecture, located in western Japan, the positive rate of serological tests for CeD was supposed to be 0.19% [5], suggesting that the presence of CeD is quite low in Japan. Due to changes in dietary habits, wheat intake has been increasing compared to the traditional rice-based diet in Japan, and wheat consumption was 4,353 g/year to 1,571 g/year in 2023 in Japan, depending on the prefecture (https://preran47.com/komugikonosyohiryo/). However, environmental factors, including gluten consumption, differ among regions. Thus, geographical variations may exist in the prevalence of CeD within Japan. Some studies have shown a strong correlation between gluten sensitivity and irritable bowel syndrome (IBS), indicating that 15% of IBS patients are subsequently diagnosed with CeD [6]. Generally, the estimated prevalence of CeD among patients diagnosed with IBS is 4 to 7 times higher than in the general population [7, 8]. In this study, we selected individuals screened for IBS or functional dyspepsia (FD) as the patient population at high risk for CeD from Hokkaido (northern Japan) to Kyushu (southern Japan). We aimed to estimate the biopsy-confirmed serofrequency of CeD among Japanese who are at a higher risk for CeD and to discern regional differences, if any.

## Patients and Methods

### Protocol and Patients

This study was performed in Japan as a part of the “Prevalence of Serologically Defined Celiac Disease in Patients with Irritable Bowel Syndrome in Asia: A Multi-Country Hospital-Based Study” [9], including six Asian countries, namely, Japan (East Asia), Indonesia, Thailand, Singapore, Malaysia (All South-East Asia), and India (South Asia). This was a hospital-based study that recruited patients with IBS, in whom the prevalence of CeD was higher than that in the general population.

Adult patients (>18 years of age, both sexes) fulfilling the criteria for IBS as defined using the Rome III criteria [10] or FD as defined using the Rome IV criteria [11] were recruited from the outpatient departments of the participating 23 centers in Japan. Participants with IBS were further subclassified according to the Rome III questionnaire into constipation-predominant IBS, diarrhea-predominant IBS (IBS-D), mixed IBS, and un-subtyped IBS [10]. Demographic data were collected at each local center. Patients with inflammatory bowel disease, tropical sprue, previous gastrointestinal surgery, other gastrointestinal diseases, allergies, or pregnancy were excluded.

### Serological Screening and Further Examination

A 5-mL blood sample was collected from each participant for complete blood counts and screening assays. The serum was separated using centrifugation at 3,000 rpm for 5 min, transferred to 1.5-mL tubes, and stored at −80°C. All blood samples were screened for anti-TTG Ab by performing fluorescence enzyme-linked immunoassay by Thermo Fisher Diagnostiscs K.K. using EliA™ Celikey^®^ IgA FEIA (Phadia AB, Thermo Fisher Scientific, Uppsala, Sweden) [12]. Test values below 10.0 U/mL were considered negative, and values >10.0 U/mL were considered positive.

Individuals testing positive for anti-TTG were counselled about the probability of having CeD and therefore requiring duodenal biopsies. Individuals who gave written and informed consent for esophagogastroduodenoscopy (EGD) and duodenal biopsy underwent the EGD to obtain duodenal biopsies; an experienced gastroenterologist performed the EGDs using an upper gastrointestinal endoscope (GIF H290; Olympus Optical Co., Ltd., Tokyo, Japan) [9, 10]. The duodenal biopsies were transferred to 10% formalin, oriented, and processed in paraffin; the hematoxylin and eosin-stained sections were examined by experienced pathologists at the respective centers. The mucosal changes were graded according to the modified Marsh classification for CeD [13].

### Regional Distribution

Japan was divided into 7 regions: Hokkaido, Tohoku, Kanto, Chubu, Kinki, Chugoku-Shikoku, and Kyushu, from northern to southern Japan. The biopsy-confirmed serofrequency of CeD was calculated in each area to estimate regional risk factors.

### Ethics

The study was reviewed and approved by the institutional ethics boards of all participating centers, with the following approval references from the supervising institute, National Defense Medical College (original no. 4379, revised no. 4846). All adult participants provided written informed consent to participate in this study.

### Statistics

A scatter plot of TTG Ab values was generated. The average values were compared among the regions. Since the value of detection sensitivity was 0.4 U/mL, values below 0.4 were regarded as 0.2 U/mL in average.

## Results

### Baseline Characteristics

A total of 583 subjects were enrolled in this study (male: 242, female: 341), including 132 with constipation-predominant IBS (male: 39, female: 93), 357 with IBS-D (male: 171, female: 186), 71 with mixed IBS (male: 26, female: 45), 2 with un-subtyped IBS (male: 0, female: 2), and 21 with FD (male: 6, female: 15) (Table 1). The average age was 44.7 ± 16.3 (16–87). The demographic details are provided in Table 1. Only 1 patient had positive TTG (16.8 IU/mL): a 26-year-old woman in the IBS-D group.

**Table 1.** Demographic details

DGBI	*N*	Male	Female	TTG >10
Total	583	242	341	0
IBS-C	132	39	93	0
IBS-D	357	171	186	1
IBS-M	71	26	45	0
IBS-U	2	0	2	0
FD	21	6	15	0
Age	44.7±16.3	45.5±16.9	44.1±9.9	26
Range	16∼87	16∼86	16∼87	–

Age was demonstrated as average ± standard deviation.

DGBI, disorders of gut-brain interaction; IBS-C, constipation-predominant IBS; IBS-D, diarrhea-predominant IBS; IBS-M, mixed IBS; IBS-U, un-subtyped IBS; FD, functional dyspepsia.

### Estimated Biopsy-Confirmed Serofrequency of CeD in Disorders of Gut-Brain Interaction

Figure 1 shows the distribution of the TTG values. Only one subject with TTG >10 was evaluated for histopathology. The patient was diagnosed with Marsh 3a CeD. The biopsy-confirmed serofrequency of CeD in gut-brain interaction (DGBI) was estimated to be 0.17% (1/583). The biopsy-confirmed serofrequency of CeD in IBS-D was estimated to be 0.28% (1/357).

**Fig. 1.**
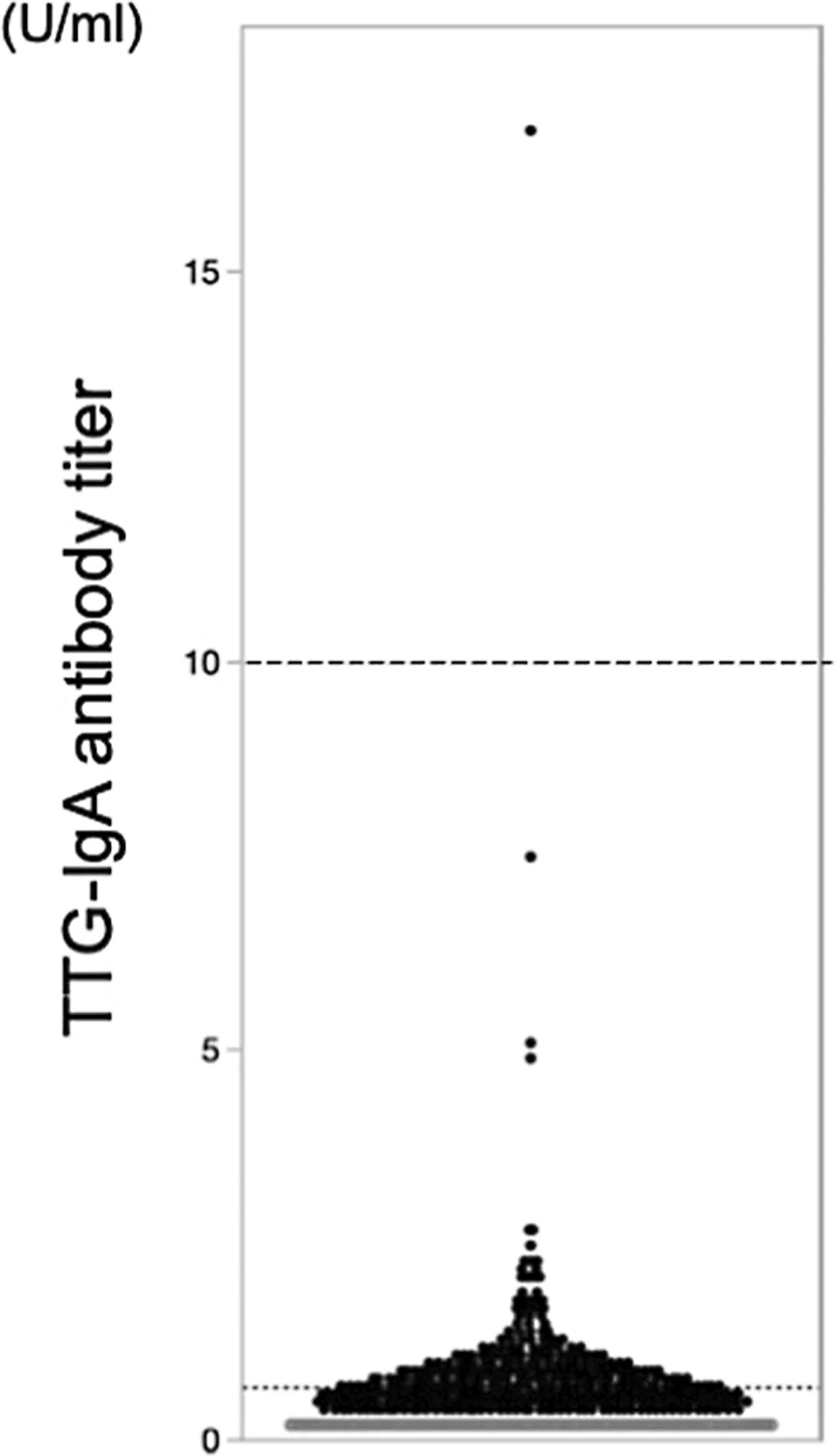
Distribution of the value of anti-tissue transglutaminase IgA (TTG-IgA). Cutoff is shown by the dashed line (10 IU/mL). Average of TTG is shown by the dotted line.

### Regional Distribution

There were 83 participants from Hokkaido, 28 from Tohoku, 264 from Kanto, 30 from Chubu, 28 from Kinki, 137 from Chugoku-Shikoku, and 14 from Kyushu (Table 2). Figure 2 shows the scatter plot of the TTG for each area. There were no significant differences among regions in each disease subset. Since there was only one subject with CeD who was enrolled in Kanagawa Prefecture (Kanto area), we could not estimate the regional risk factors in each area.

**Table 2.** Demographic details in each area

DGBI	Hokkaido	Tohoku	Kanto	Chubu	Kinki	Chugoku-Shikoku	Kyushu
Total	83	28	263	30	28	137	14
IBS-C	34	1	45	5	1	46	0
IBS-D	36	20	170	23	20	74	14
IBS-M	1	7	37	2	7	17	0
IBS-U	0	0	2	0	0	0	0
FD	12	0	9	0	0	0	0
Age	38.9±9.9	45.6±13.3	46.1±16.7	40.1±15.5	41.2±11.3	48.3±19.5	38.0±10.8
Range	18∼50	21∼81	16∼83	16∼73	21∼73	16∼87	20∼63

Age was demonstrated as average ± standard deviation.

DGBI, disorders of gut-brain interaction; IBS-C, constipation-predominant IBS; IBS-D, diarrhea-predominant IBS; IBS-M, mixed IBS; IBS-U, un-subtyped IBS; FD, functional dyspepsia.

**Fig. 2.**
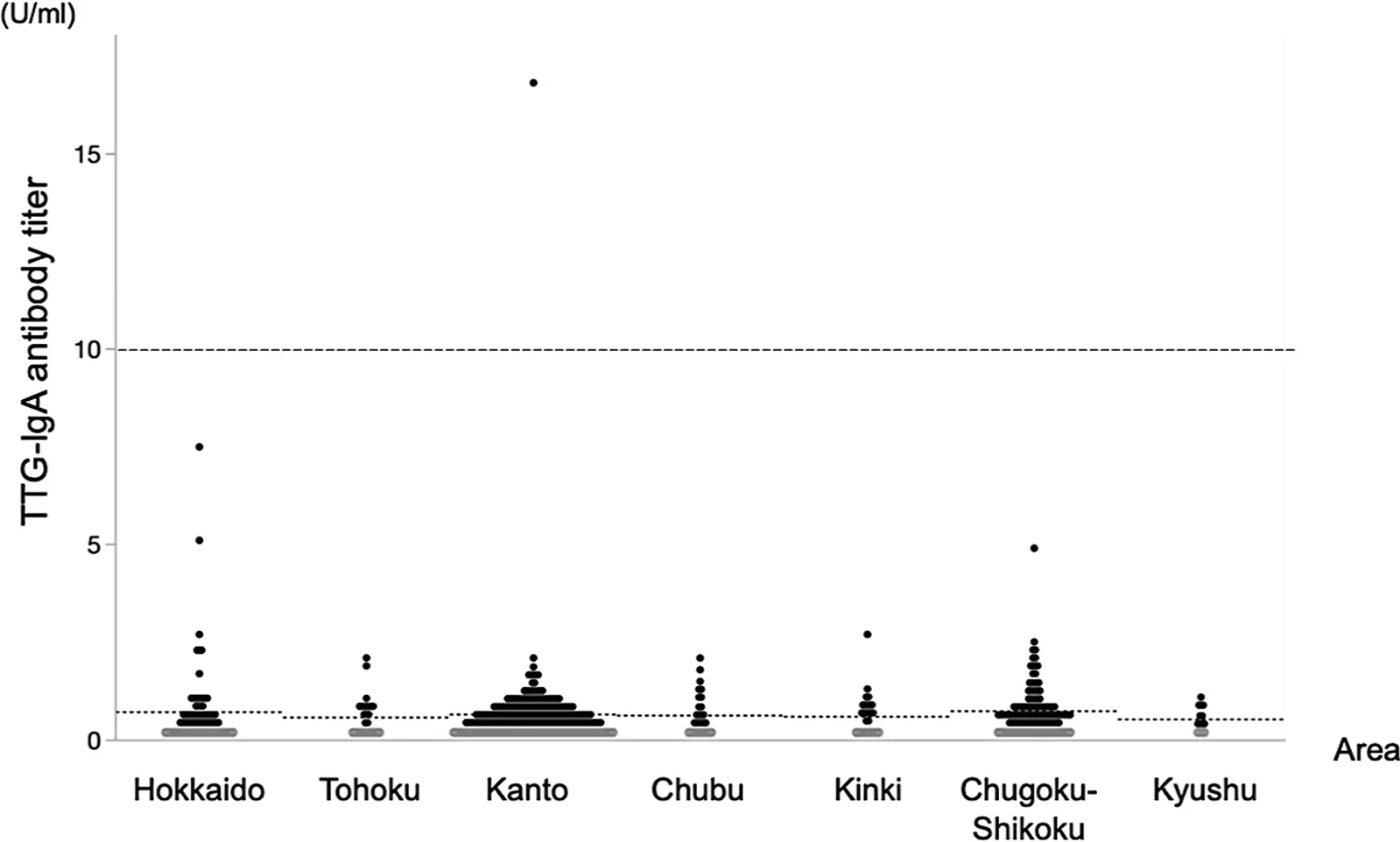
Distribution of the value of anti-tissue transglutaminase IgA (TTG-IgA) in each area from Hokkaido area to Kyushu area. Cutoff is shown by the dashed line (10 IU/mL). Average is shown by the dotted line.

## Discussion

Until a few decades prior, CeD was considered an uncommon disease affecting mainly children and limited to Western Europe [14]. Over the past 2 decades, CeD has emerged as a major public health concern worldwide. Initial studies on its prevalence in the general population were conducted in European countries, and it has been estimated that CeD affects approximately 1% of the European population [5, 6]. CeD was subsequently reported in other parts of the world with predominantly Caucasian populations such as North America and Australia as well as in some South American countries such as Brazil [7–9]. Recently, population-based data on the prevalence of CeD have been reported in Middle Eastern Asia, India, and several African countries [10, 11]. However, CeD was supposed to be rare in Japan until now. In a Chinese study involving 246 patients with IBS-D, 2.85% had CeD [15]. Therefore, we hypothesized that there was a possibility that some cases were overlooked among patients with DGBI, especially those with diarrheal symptoms, because of a lack of awareness. In the present study, the biopsy-confirmed serofrequency was approximately 0.3% among patients with IBS-D in several regions, suggesting that an increase in the prevalence of CeD has not yet been observed in any region of Japan. The rarity of CeD appears to be a nationwide characteristic of Japanese IBS patients rather than a localized phenomenon. In this study, our observed frequency (0.17%) is numerically similar to prior reports on the general healthy population in Japan (approx. 0.05–0.1%). Our study is the first large-scale, multicenter effort to show that in Japan, IBS patients do not exhibit an increased frequency of CeD compared to the general population. This provides a vital message for Japanese clinicians: routine, universal screening for CeD in patients presenting with IBS symptoms may be clinically inefficient in Japan.

Type 1 diabetes mellitus (T1DM) is an autoimmune disorder; patients with T1DM have an increased risk of developing other autoimmune diseases, including CeD [16]. According to a review, the prevalence of CeD among patients with T1DM based on serological tests was 1.4%–24.5% [17]. However, in Japan, the prevalence of CeD among patients with T1DM was found to be 0% [18], suggesting that the prevalence of CeD is very low compared to that in other countries, even among high-risk patients with T1DM, although this was a single-center study. The differences in halotypes associated with genetic susceptibility to T1DM are attributed to differences in the prevalence of CeD in T1DM according to race [18]. The 1 patient with CeD in our study did not have T1DM.

In our study, since we did not assess actual gluten intake for each subject, there is a possibility that some patients might have reduced their gluten intake due to abdominal symptoms associated with gluten consumption**.** Therefore, one limitation of our study is the lack of data on gluten consumption. There is another limitation to this study: serum IgA levels were not examined. It has been reported that the incidence of IgA deficiency in patients with CeD ranges from 2% to 3%, indicating a 10- to 15-fold increase compared to individuals with normal IgA levels [19]. While the prevalence of IgA deficiency is remarkably low in Japan (0.007%) [20], the measurement of serum IgA levels allows for a more accurate assessment of CeD prevalence. Thus, true prevalence cannot be established without detailed data on individual gluten intake and total IgA levels. Our sample size was severely underpowered to evaluate regional variations. However, we demonstrate that CeD is extremely rare among Japanese IBS patients, contrary to Western guidelines recommending routine screening. Although the sample size of approximately 600 was initially calculated based on a different clinical assumption, a post hoc power analysis demonstrated that this cohort provided sufficient statistical power to test our primary descriptive hypothesis. Specifically, assuming a reference CeD frequency of 2.0%–3.0% as reported in Western IBS cohorts, a sample size of 600 achieves 95.4% to >99.8% power (one-sample binomial test, two-sided, alpha = 0.05) to confirm that the observed frequency in our Japanese cohort is significantly lower. Thus, the sample size was statistically robust enough to conclude the clinical rarity of CeD in Japanese patients with IBS, despite the wide confidence interval for the exact point estimate.

In addition, the study population, predominantly from university-based hospitals, and small sample size were other limitations of this study. Regarding geographic distribution, the single patient with biopsy-confirmed CeD was identified in the Kanto region. Due to the occurrence of only one positive case across the entire cohort, statistical analysis for regional variation was not feasible. Nevertheless, our study recruited patients from multiple centers across various regions nationwide. The fact that only one case was detected in this nationwide cohort descriptively suggests that the rarity of CeD among Japanese IBS patients is likely a widespread phenomenon rather than a localized trend, although larger, country-wide registries would be required to confirm true geographic uniformity.

## Conclusion

It is noteworthy that no clustering of CeD cases in Japanese patients with DGBI was observed, and the overall frequency remained extremely low across all participating centers nationwide.

## Acknowledgments

We acknowledge the support of all the research teams at each participating center for their kind support in the conduct of the study.

## Statement of Ethics

The study was reviewed and approved by the institutional ethics boards of all participating centers, with the following approval references from the supervising institute, National Defense Medical College (original No. 4379, revised No. 4846). All adult participants provided written informed consent to participate in this study.

## Conflict of Interest Statement

The authors declare no conflict of interest. Akira Andoh, Hiroshi Nakase, Mamoru Watanabe, and Ryota Hokari were members of the journal’s Editorial Board at the time of submission.

## Funding Sources

This study was supported by a research grant from Thermo Fisher Diagnostics K.K., Japan. The funding source provided financial support for planning the study protocol and performed the anti-TTG Ab laboratory examinations. Beyond these contributions, the funding source had no role in the study design, data execution, data analysis, or interpretation of the data. Furthermore, the funding source was not involved in the manuscript conception, planning, writing, or the decision to submit the manuscript for publication. All data from the 23 participating centers were managed and analyzed independently by the authors.

## Author Contributions

G.M., Y.K., and R.H. conceptualized and designed the study. M.H., C.K., and R.H. performed the data analysis and interpreted the results. M.H. and R.H. drafted the manuscript. RH supervised the project. Authors in participating 23 centers in Japan (M.H., A.S., T.M., M.F., A.N., S.F., H.I., E.I., K.I., S.T., T.U., Y.F., A.A., N.O., H.K., T.O., T.M., H.N., H.S., M.W., K.U., H.S., and M.N.) recruited subjects. All authors reviewed and approved the final manuscript.

## Data Availability

The data that support the findings of this study are not publicly available due to privacy reasons but are available from the corresponding authors upon request.
